# Supplementary material for: Global plant trait relationships extend to the climatic extremes of the tundra biome
Source: Nat Commun. 2020 Mar 12;11:1351. doi: 10.1038/s41467-020-15014-4 (PMC7067758; doi:10.1038/s41467-020-15014-4)
Supplement: Supplementary file 1 — Supplementary Information [file 41467_2020_15014_MOESM1_ESM.pdf]

Supplementary Materials

Below are all supplementary tables and figures supporting the analyses set out in 'Global plant trait relationships extend to the climatic extremes of the tundra biome' by Thomas et al.

1. Supplementary Figures **3**

Supplementary Figure 1: Distribution of tundra species along PCA axes

3

Supplementary Figure 2: Trait distributions and correlations

4

Supplementary Figure 3: Comparison of trait loadings for PCA axes

5

Supplementary Figure 4: Contribution of traits loadings to each PCA axis

6

Supplementary Figure 5: Trait variation above and below break-points

7

Supplementary Figure 6: Trait variation for LDMC and seed mass 8

Supplementary Figure 7: Sources of trait variation over geographic scale (non-logged)

10

Supplementary Figure 8: Sources of trait variation with species richness (non-logged)

11

Sensitivity test: leaf dry matter content to stem specific density conversion

Supplementary Figure 9: Global and tundra trait-space

12

Supplementary Figure 10: Trait distributions and correlations 13

Supplementary Figure 11: Comparison of trait loadings for PCA axes 14

Sensitivity test: minimum species and site cut-off increased from three to five

Supplementary Figure 12: Sources of trait variation over geographic scale 15

Supplementary Figure 13: Sources of trait variation with species richness 16

Sensitivity test: only trait collection sites north of 6.5°N and below 0°C

Supplementary Figure 14: Collection sites in geographic and climate space 17

Supplementary Figure 15: Global and tundra trait-space 19

Supplementary Figure 16: Trait distributions and correlations 20

Supplementary Figure 17: Comparison of trait loadings for PCA axes 21

Supplementary Figure 18: Sources of trait variation across traits 22

Supplementary Figure 19: Sources of trait variation over geographic scale 23

Supplementary Figure 20: Sources of trait variation with species richness 24

2. Supplementary Tables **25**

Supplementary Table 1: Number of observations per trait 25

3. Supplementary References **26**

Supplementary Figures

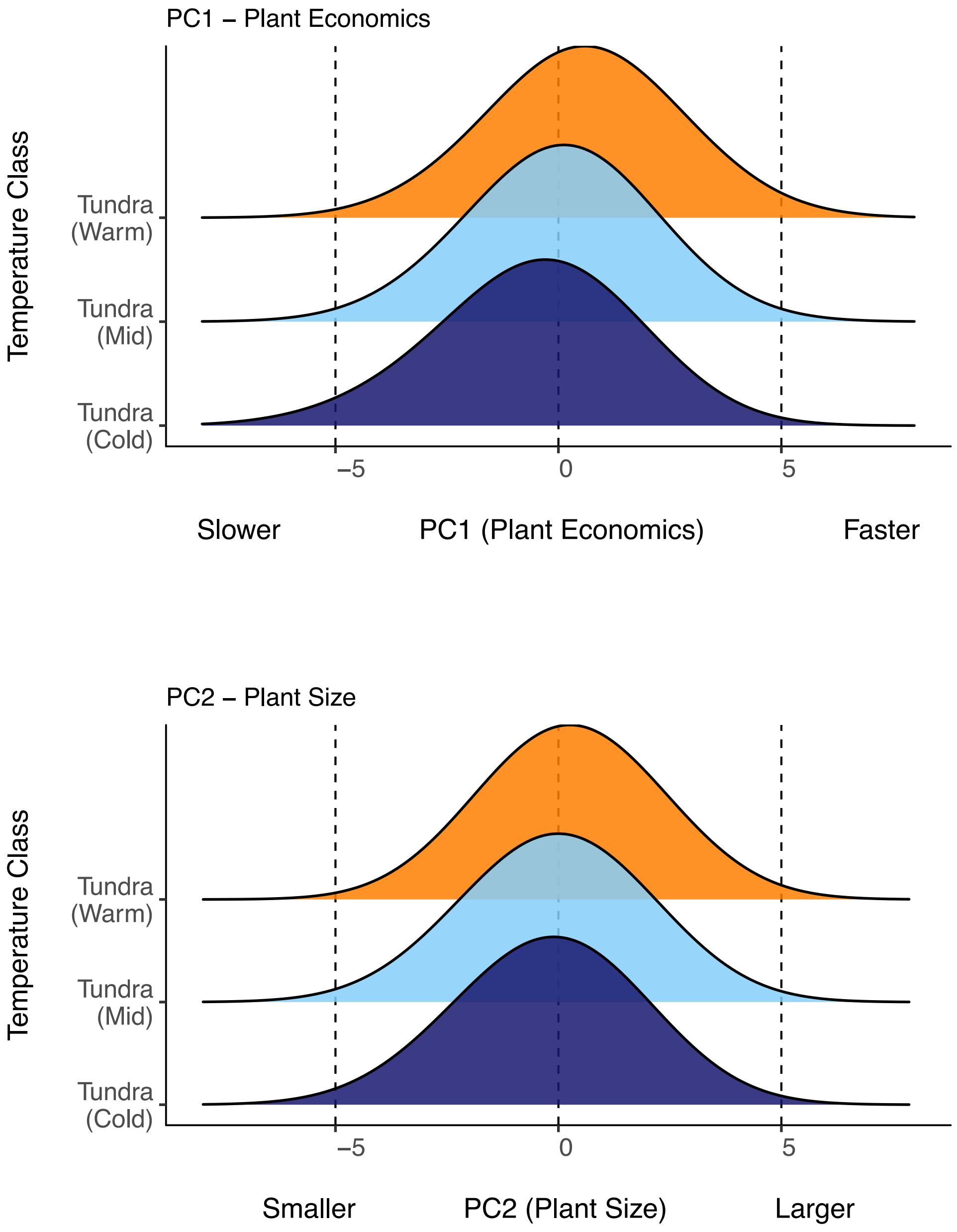

Supplementary Figure 1: Distribution of tundra species along the two dimensions of trait variation, grouped by 'temperature class' of species. Curves indicate the density distribution of species along PCA axes. Points are coloured by temperature category, corresponding to the mean annual temperature of trait collection sites for each species (Cold < –1 °C, Mid > –1 °C but <1 °C, Warm >1 °C).

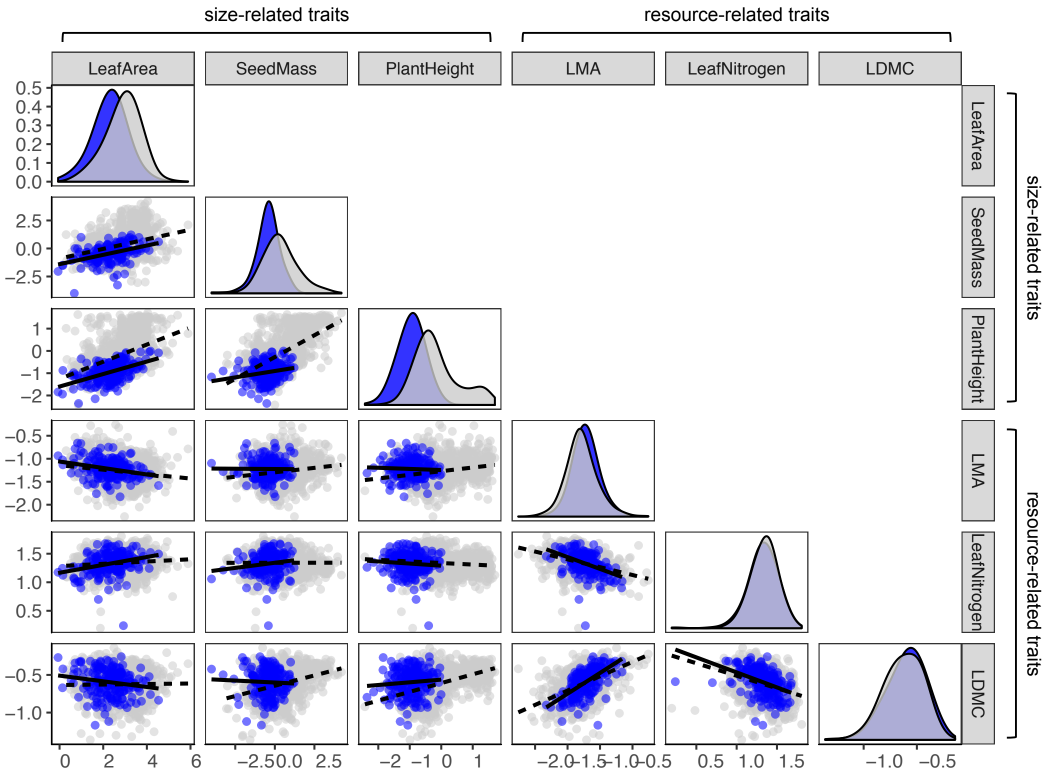

Supplementary Figure 2: Comparison of trait distributions and trait correlations among tundra (blue) and global vascular plant species (grey). Lower left panels: pairwise trait correlations among the six plant traits tested in this study (Leaf Area, Seed Mass, Plant Height, LMA – Leaf Mass per Area, Leaf Nitrogen, LDMC – Leaf Dry Matter Content). Points represent the mean value for each species. Solid lines indicate fit for tundra species, dashed lines indicate fit for global species. Diagonal panels: smoothed distribution of log transformed trait values for tundra and global plant species.

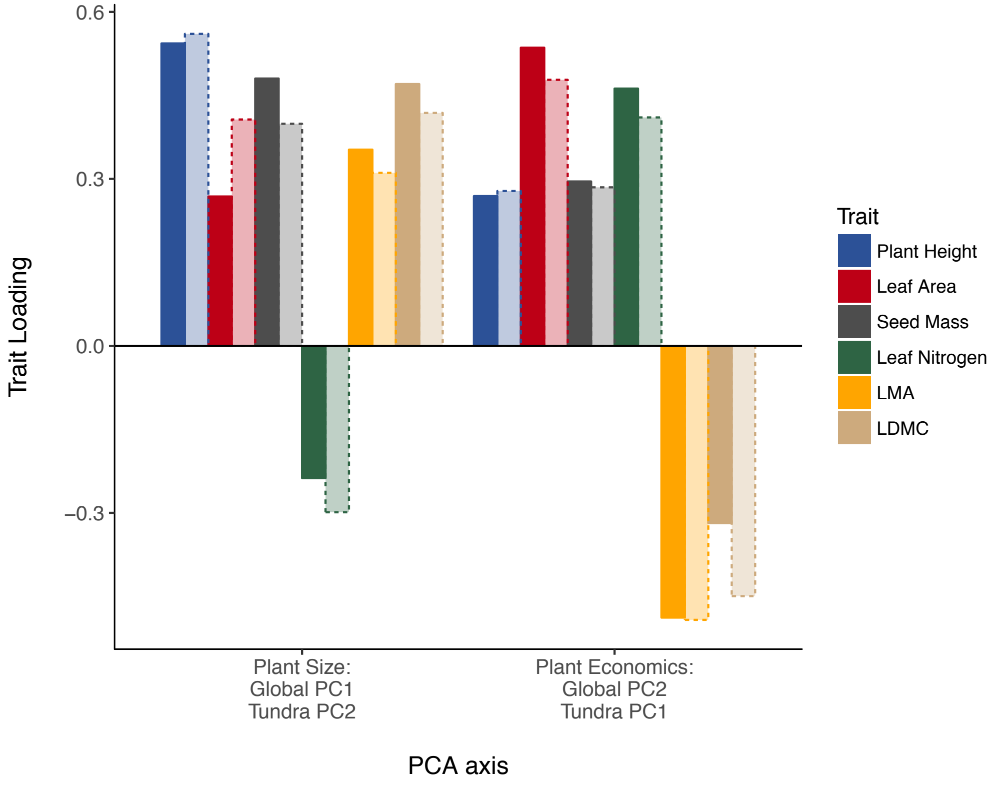

Supplementary Figure 3: Comparison of trait loadings for the first two PCA axes for tundra and global vascular plant species. Colours indicate traits (red = leaf area, brown = seed mass, green = adult plant height, light blue = leaf mass per area (LMA), dark blue = leaf nitrogen, pink = leaf dry matter content (LDMC)). Solid, darker bars indicate loadings for global species, dashed and lighter coloured bars indicate loadings for tundra species. The relative importance of PCA axes is reversed for tundra species, such that PCA 1 for global species corresponds with PCA 2 for tundra species.

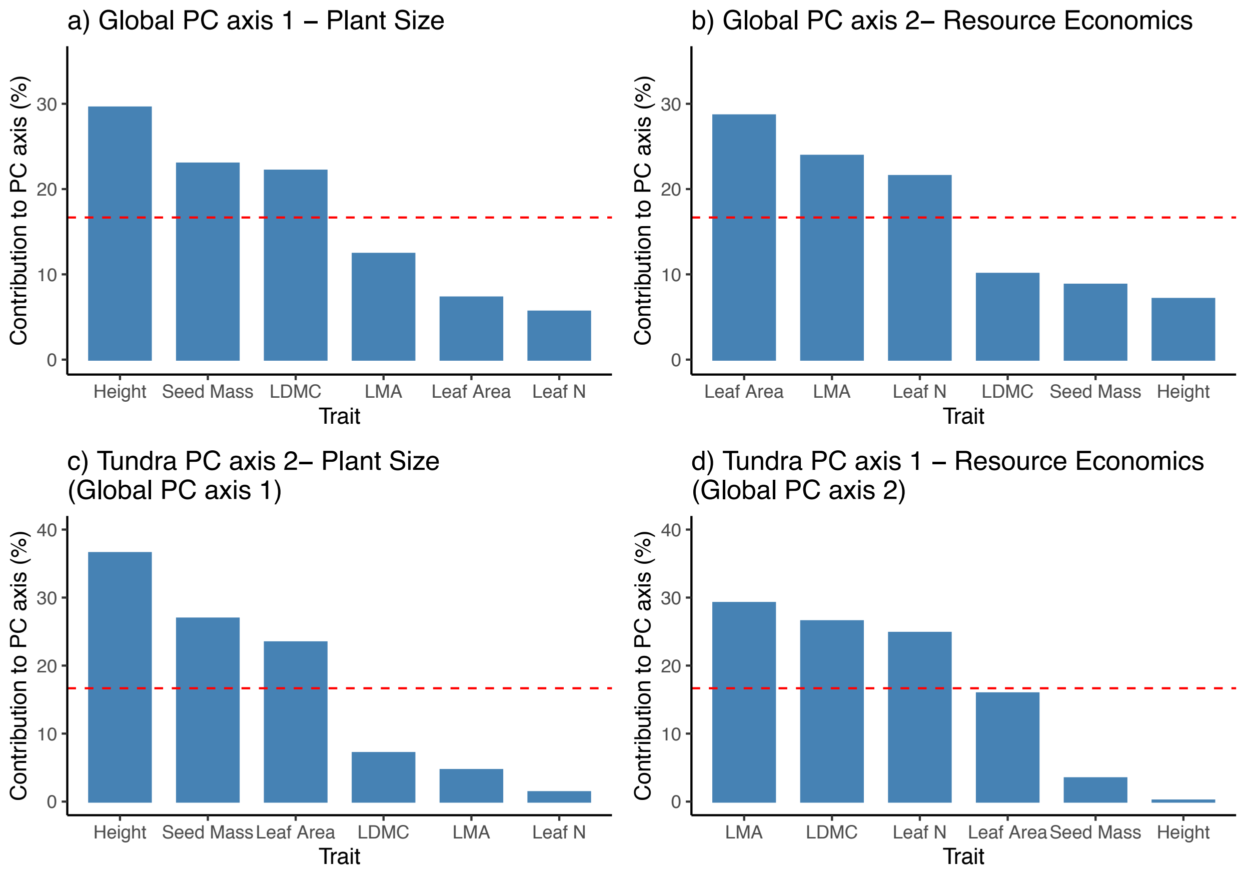

Supplementary Figure 4: Contribution of each trait loading to PCA axes. Plant height, seed mass and leaf dry matter content primarily contributed to PCA axis 1 (associated with plant size) among (a) global and (c) tundra species. LMA, leaf area and leaf dry matter content primarily contributed to PCA axis 2 (associated with resource) among (b) global and (d) tundra species. Bars indicate contribution of each trait to PCA axis; dashed red line indicates percentage contribution of all traits contributed equally (16.7%).

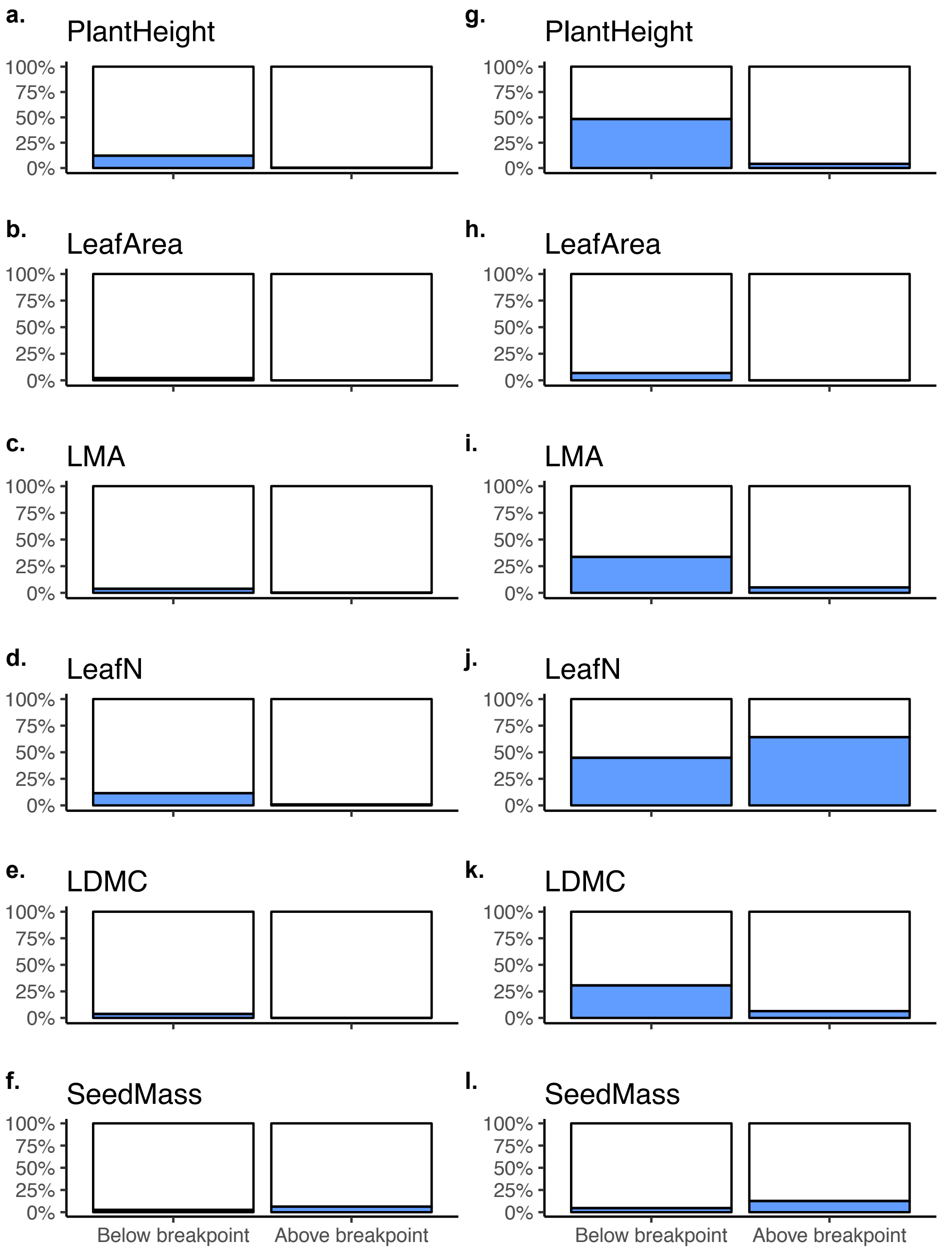

Supplementary Figure 5: Proportion of sites for which within-species variation accounts for at least half of trait variation (a-f) or at least one third of trait variation (g-l) among tundra species. Bars indicate the proportion of sites above and below the break point of change in source of variation over geographical scale (Figure 4, Supplementary Figure 6).

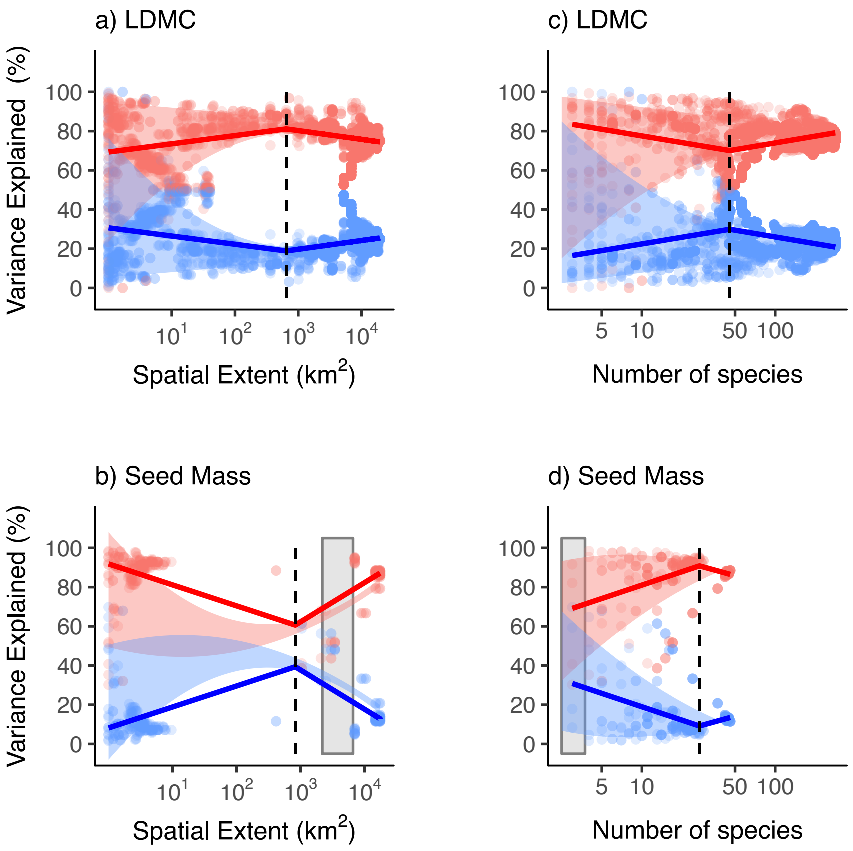

Supplementary Figure 6: Sources of trait variation across geographical scale (a-b) and species richness (c-d) for leaf dry matter content, and seed mass (plant height, leaf area, leaf mass per area and leaf nitrogen shown main text, Fig. 4). Variance partitioning occurs over incrementally increasing sampling steps, beginning at a given trait collection site and iteratively adding sites based on geographical proximity until the whole biome is represented. The process is repeated for every trait-sampling site. Points indicate contribution to total trait variation from between-species variation (red) and within-species variation (blue) at every sampling step. Coloured lines indicate linear break point model fit with one break point and black dashed line indicates break point location. Error bounds indicate 95% of variation across sampling sites. Grey box indicates where the difference between among- and within-species richness is not significant (two-tailed t test; $P > 0.05$). The x-axis in (a-b) represents trait variation over geographic scale, where scale represents the maximum distance from the initial sampling site,

presented on a log10 scale. The x-axis in (c-d) represents trait variation with species richness, presented on a log10 scale.

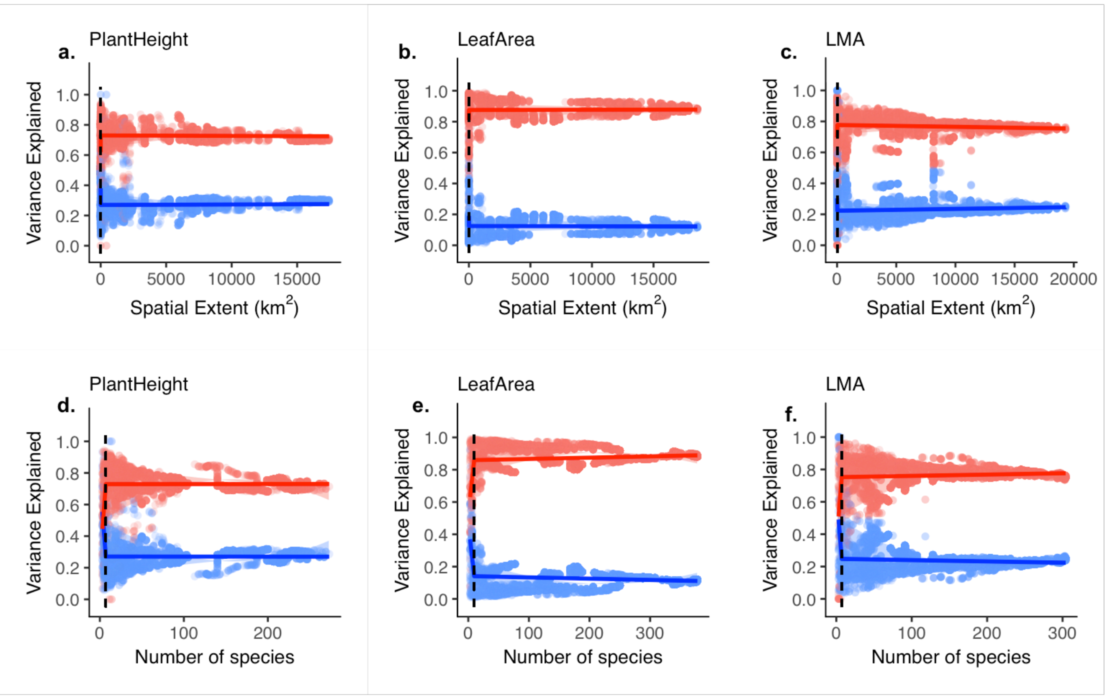

Supplementary Figure 7: Sources of trait variation across non-log_{10} transformed geographical scale for leaf area, plant height, leaf mass per area, leaf nitrogen, leaf dry matter content and seed mass (as opposed to log_{10} transformed geographical scale in Fig. 4 in the main text). Variance partitioning occurs over incrementally increasing sampling steps, beginning at a given trait collection site (first step) and iteratively adding sites based on geographical proximity until the whole biome is represented (final step). The process is repeated for every trait-sampling site. Points indicate contribution to total trait variation from between-species variation (red) and within-species variation (blue) at every sampling step. Coloured lines indicate linear break point model fits with one break point. Dashed line indicates break point location. Error bounds indicate 95% of variation across sampling sites.

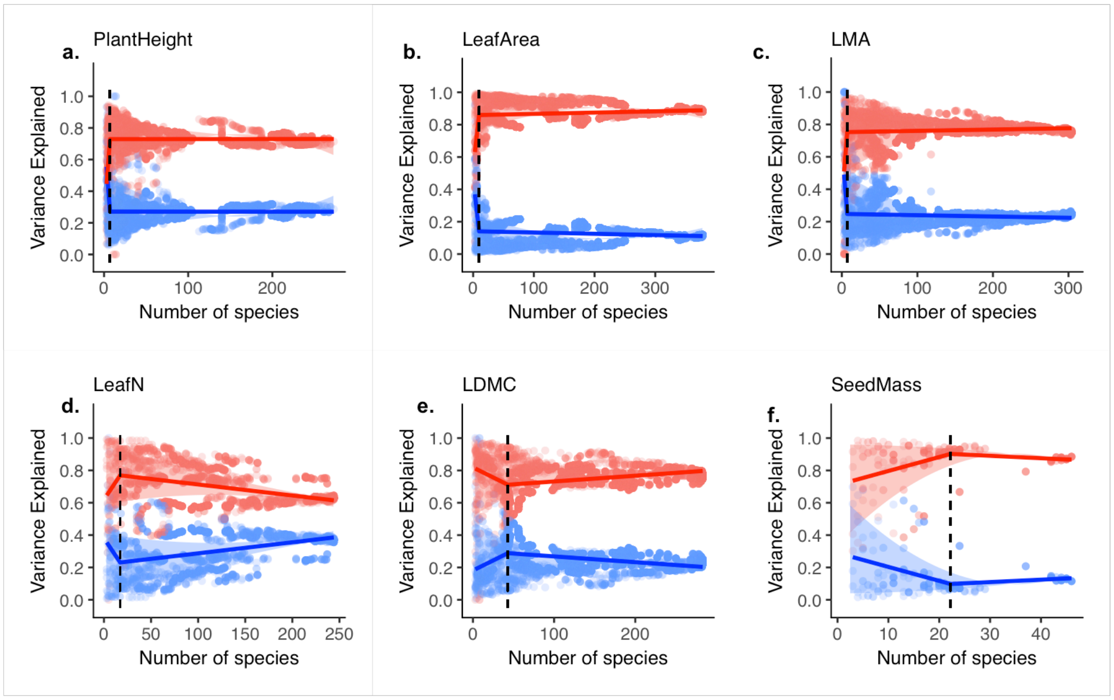

Supplementary Figure 8: Sources of trait variation with non-$\log_{10}$ transformed species richness for leaf area, plant height, leaf mass per area, leaf nitrogen, leaf dry matter content and seed mass (as opposed to $\log_{10}$ transformed species richness in Fig. 4 in the main text). Variance partitioning occurs over incrementally increasing sampling steps, beginning at a given trait collection site (first step) and iteratively adding sites based on geographical proximity until the whole biome is represented (final step). The process is repeated for every trait-sampling site. Points indicate contribution to total trait variation from between-species variation (red) and within-species variation (blue) at every sampling step. Coloured lines indicate linear break point model fits with one break point. Dashed line indicates break point location. Error bounds indicate 95% of variation across sampling sites. Grey shaded area indicates where differences in the contribution of within-species and among-species variation to overall trait variation are not significant (two-tailed t test, $P > 0.05$).

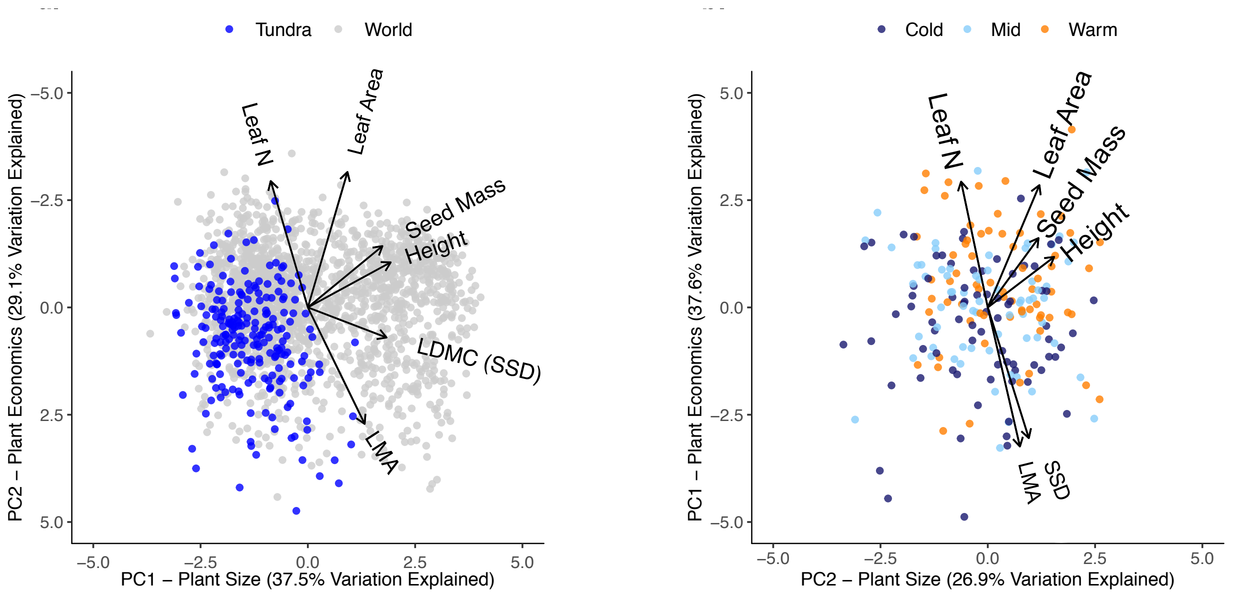

Supplementary Figure 9: Distribution of trait-space as defined by six plant traits as in Fig. 2, but with the inclusion of trait values converted from specific stem density to leaf dry matter content based on trait co-variation. Height = adult plant height, LMA = leaf mass per area, Leaf N = leaf nitrogen, LDMC = leaf dry matter content. (a) Location of tundra species within global trait-space, following [1]. Points represent mean trait values for each of global plant species defined by two component axes. Grey points represent 1744 plant species in the global TRY dataset; blue points represent 220 tundra species. Arrows indicate the direction and weighting of trait vectors for all observations. (b) Distribution of trait space for tundra species only. Points represent mean trait values for tundra species, coloured by 'temperature class'. Temperature class corresponds to the mean annual temperature of trait collection sites for each species (Group 1 – cold tundra: < -1°C, Group 2 – mid tundra: < 1°C, Group 3 – warm tundra: > 1 °C). Arrows represent direction and magnitude of trait loadings.

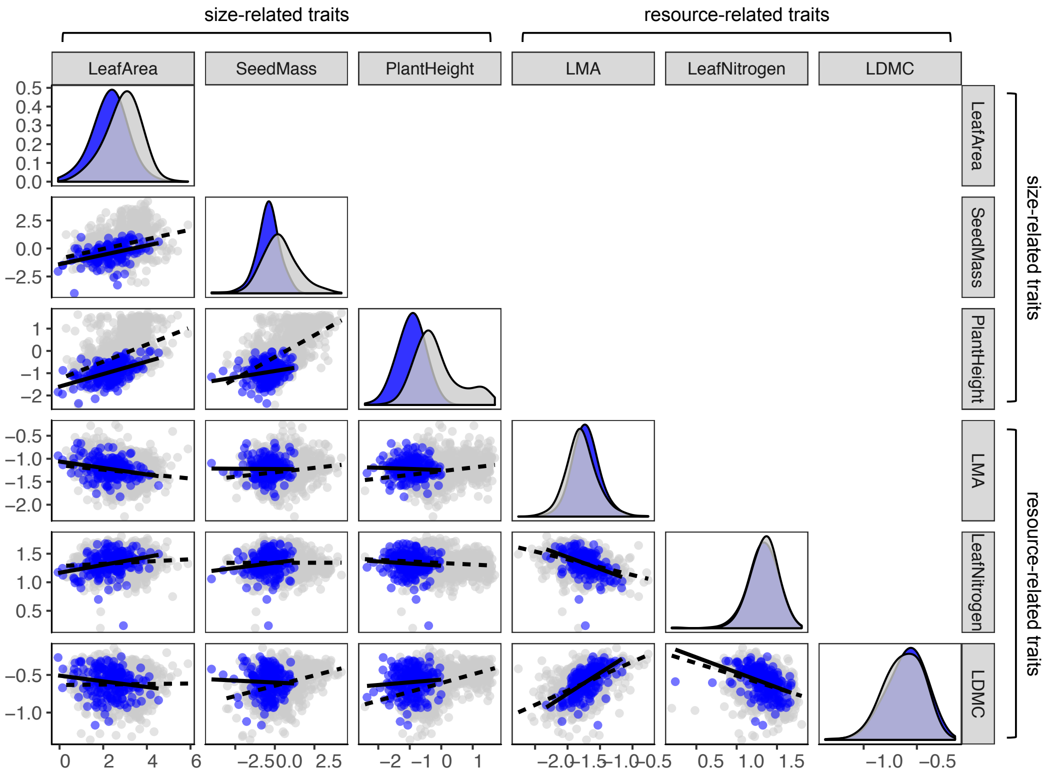

Supplementary Figure 10: Comparison of trait distributions and trait correlations among tundra (blue) and global vascular plant species (grey) as in Supplementary Figure 2, but with the inclusion of trait values converted from specific stem density to leaf dry matter content based on trait co-variation. Lower left panels: pairwise trait correlations among the six plant traits tested in this study (Leaf Area, Seed Mass, Plant Height, LMA – Leaf Mass per Area, Leaf Nitrogen, LDMC – Leaf Dry Matter Content). Points represent the mean value for each species. Solid lines indicate fit for tundra species, dashed lines indicate fit for global species. Diagonal panels: smoothed distribution of log transformed trait values for tundra and global plant species.

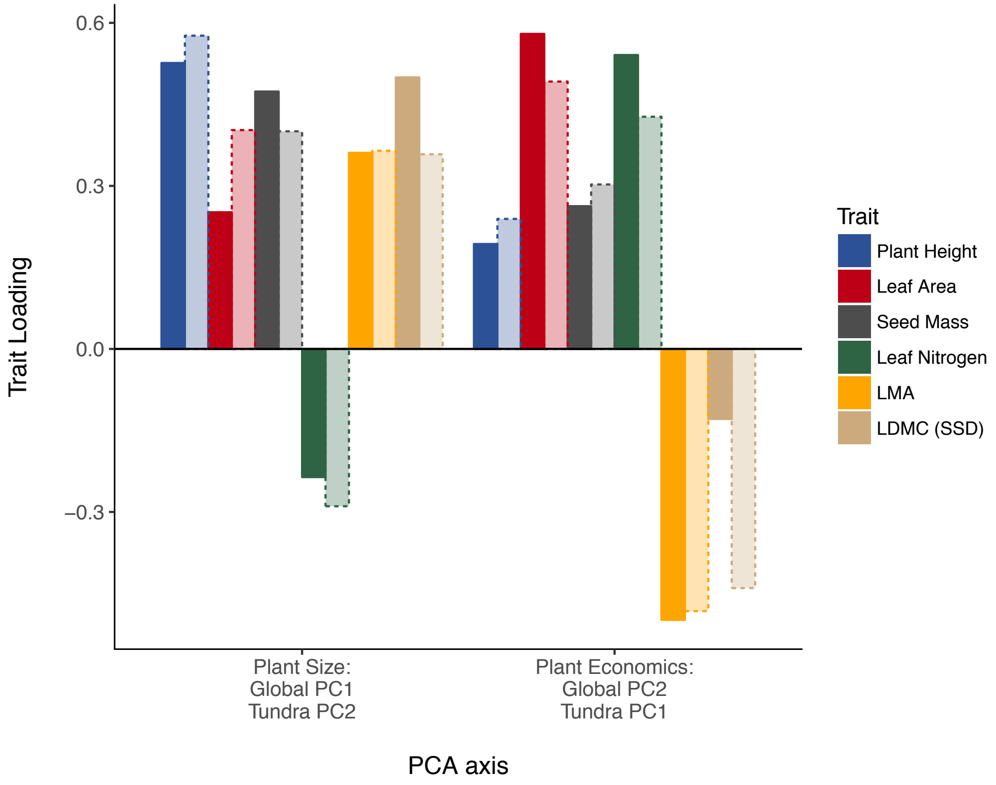

Supplementary Figure 11: Comparison of trait loadings for the first two PCA axes for tundra and global vascular plant species as in Supplementary Figure 3, but with the inclusion of trait values converted from specific stem density to leaf dry matter content based on trait co-variation. Colour indicates trait (red = leaf area, brown = seed mass, green = adult plant height, light blue = LMA – leaf mass per area, dark blue = leaf nitrogen, pink = leaf dry matter content). Solid bars indicate loadings for global species, dashed and lighter coloured bars indicated loadings for tundra species. The relative importance of PCA axes is reverse for tundra species, such that PCA 1 for global species corresponds with PCA 2 for tundra species.

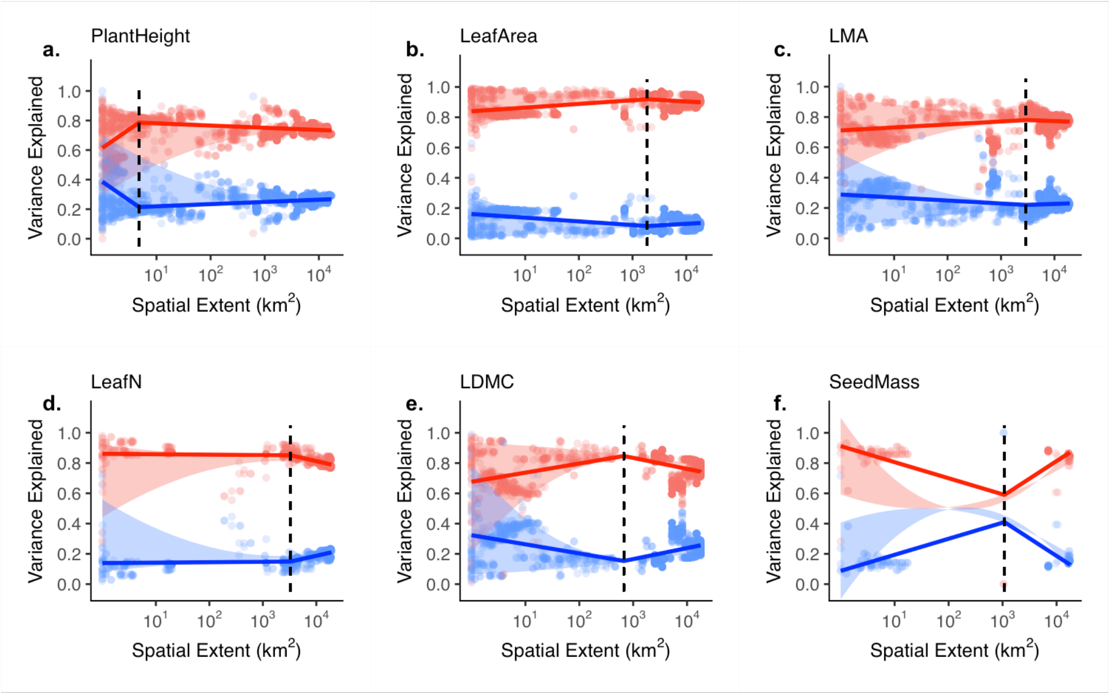

Supplementary Figure 12: Sources of trait variation across geographical scale as in Fig. 4 and Supplementary Figure 6, but excluding sites with fewer than five species or five trait observations per species, as opposed to three species or trait observations in the main text. Points indicate contribution of within-species (blue) and among-species (red) variation to total trait variation across trait sampling site combinations. Coloured lines indicate linear break point model fit with one break point. Dashed line indicates break point location.

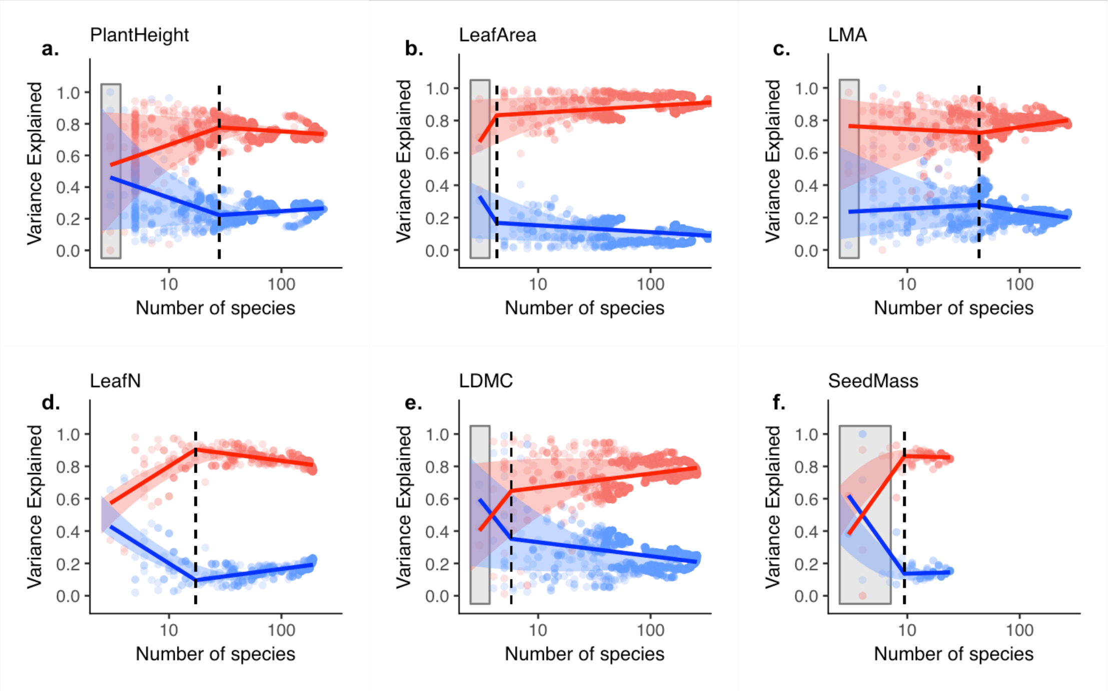

Supplementary Figure 13: Sources of trait variation across species richness as in Fig. 4 and Supplementary Figure 3, but excluding sites with fewer than five species or five trait observations per species, as opposed to three species or trait observations in the main text. Points indicate contribution of within-species (blue) and among-species (red) variation to total trait variation across trait sampling site combinations. Coloured lines indicate linear break point model fit with one break point. Dashed line indicates break point location. Grey box indicates where difference between among- and within-species variation is not significant (two-tailed t test, $P > 0.05$).

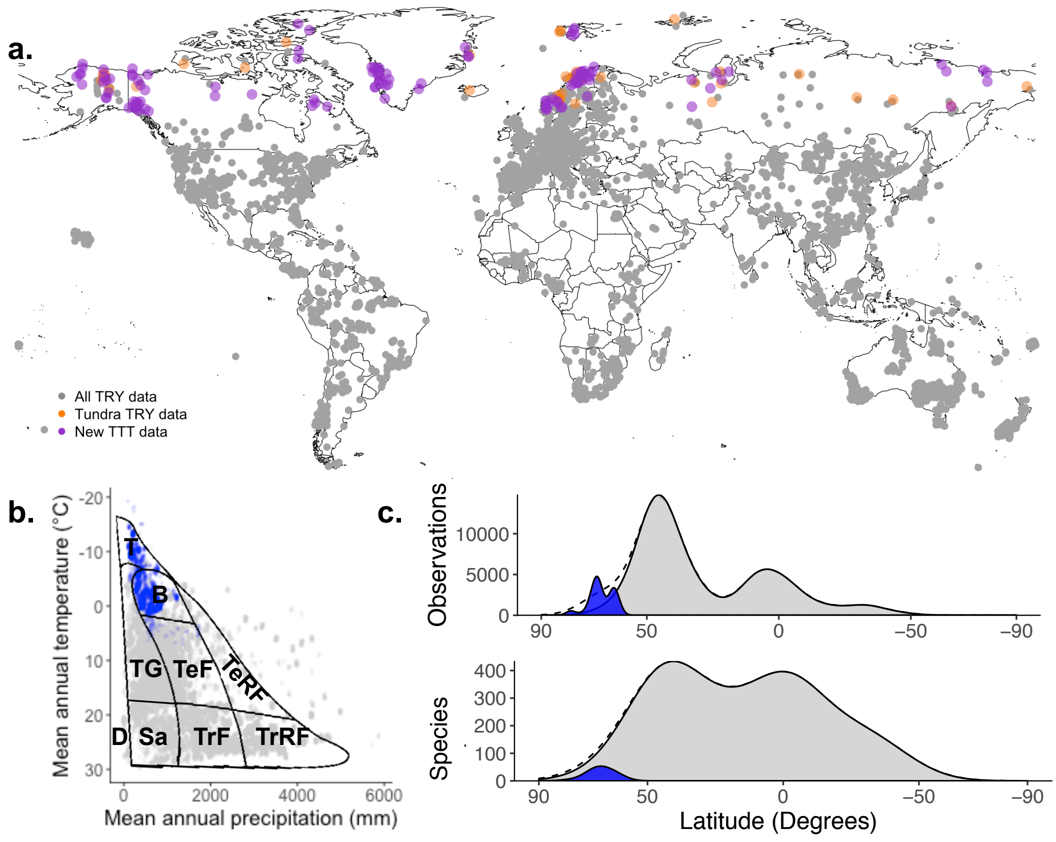

Supplementary Figure 14: Distribution of trait data for tundra species as in Fig. 1, but using only trait observations collected north of the Arctic Circle (66.5°N) or from collection locations with a mean annual temperature below 0°C. (a) Map of trait observation sites for six traits (plant height, leaf area, leaf mass per area, leaf nitrogen, leaf dry matter content, seed mass), indicating all species in the TRY database[2] (grey points), tundra species used in this analysis in the TRY database (orange points) and in new TTT data (Tundra Trait Team; purple points). (b) Location of tundra species observation sites in climate space. Mean annual temperature and mean annual precipitation are based on CHELSA climate data. Tundra species observation sites (TRY & TTT) are represented by blue points, observation points for global species are indicated by grey points. Major global biomes are mapped onto climate space (adapted from [[2]]):

T - Tundra; B - Boreal Forest; TG - Temperate Grassland; TeF - Temperate Deciduous Forest; TeRF - Temperate Rain Forest; TrF - Tropical Deciduous Forest; TrRF - Tropical Rain Forest; Sa - Savanna; D - Desert. (c) Comparison of the distribution of number of observations (upper panel) for all six traits and number of species (lower panel) in the tundra biome (blue curve) with latitude, compared to all global species in the TRY database (grey curve). The dotted curve indicates the new global distribution with the inclusion of new TTT collected data.

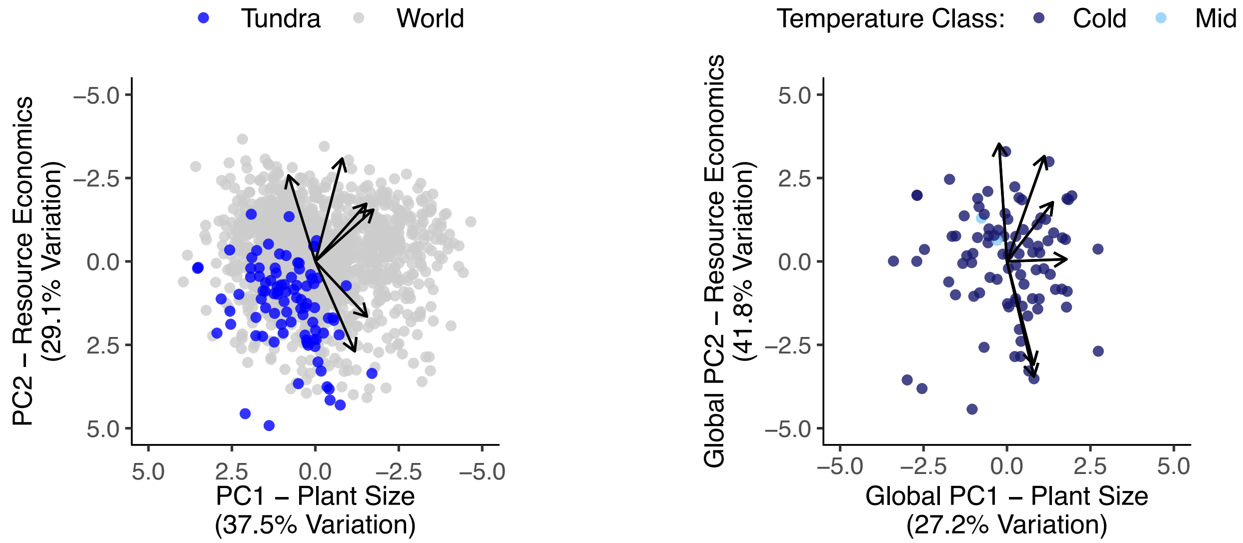

Supplementary Figure 15: Distribution of trait-space as defined by six plant traits as in Fig. 2, but only trait observations collected north of the Arctic Circle (66.5°N) or from collection locations with a mean annual temperature below 0°C. Height = adult plant height, LMA = leaf mass per area, Leaf N = leaf nitrogen, LDMC = leaf dry matter content. (a) Location of tundra species within global trait-space, following [1]. Points represent mean trait values for global plant species (including tundra species) defined by two component axes. Grey points represent plant species in a global dataset; blue points represent tundra species. Arrows indicate the direction and weighting of trait vectors. (b) Distribution of trait space for tundra species only. PCA Points represent mean trait values for 57 tundra species, coloured by 'temperature class'. Points are coloured by temperature category, corresponding to the mean annual temperature of trait collection sites for each species (Cold < −1 °C, Mid > −1 °C but <1 °C, Warm >1 °C). Arrows represent direction and magnitude of trait loadings.

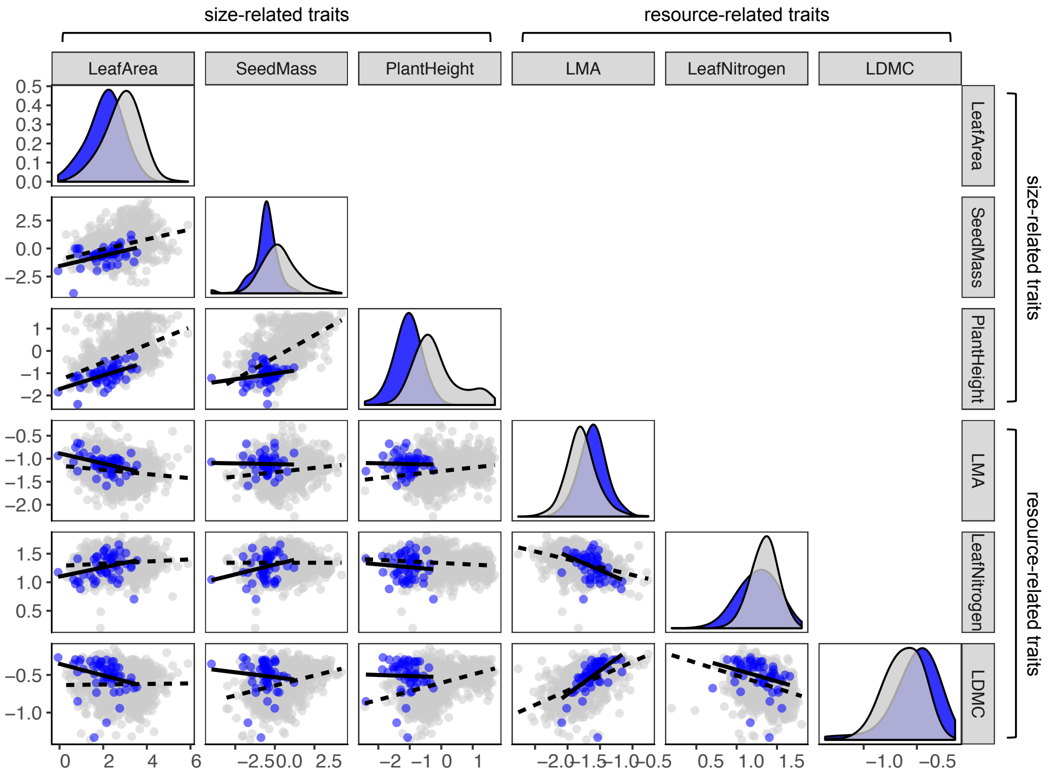

Supplementary Figure 16: Comparison of trait distributions and trait correlations among tundra (blue) and global vascular plant species (grey) as in Supplementary Figure 2, but only trait observations collected north of the Arctic Circle (66.5°N) or from collection locations with a mean annual temperature below 0°C. Lower left panels: pairwise trait correlations among the six plant traits tested in this study (Leaf Area, Seed Mass, Plant Height, LMA – Leaf Mass per Area, Leaf Nitrogen, LDMC – Leaf Dry Matter Content). Points represent the mean value for each species. Solid lines indicate fit for tundra species, dashed lines indicate fit for global species. Diagonal panels: smoothed distribution of log transformed trait values for tundra and global plant species.

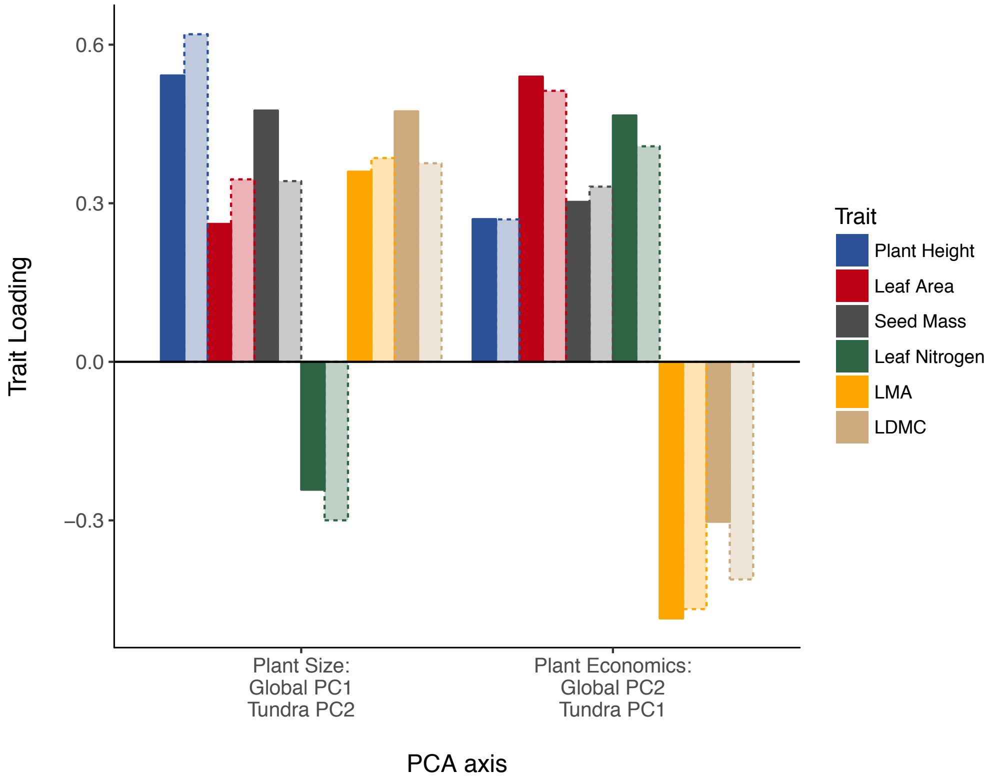

Supplementary Figure 17: Comparison of trait loadings for the first two PCA axes for tundra and global vascular plant species as in Supplementary Figure 3, but only trait observations collected north of the Arctic Circle (66.5°N) or from collection locations with a mean annual temperature below 0°C. Colour indicates trait (red = leaf area, brown = seed mass, green = adult plant height, light blue = LMA – leaf mass per area, dark blue = leaf nitrogen, pink = leaf dry matter content). Solid bars indicate loadings for global species, dashed and lighter coloured bars indicated loadings for tundra species. The relative importance of PCA axes is reverse for tundra species, such that PCA 1 for global species corresponds with PCA 2 for tundra species.

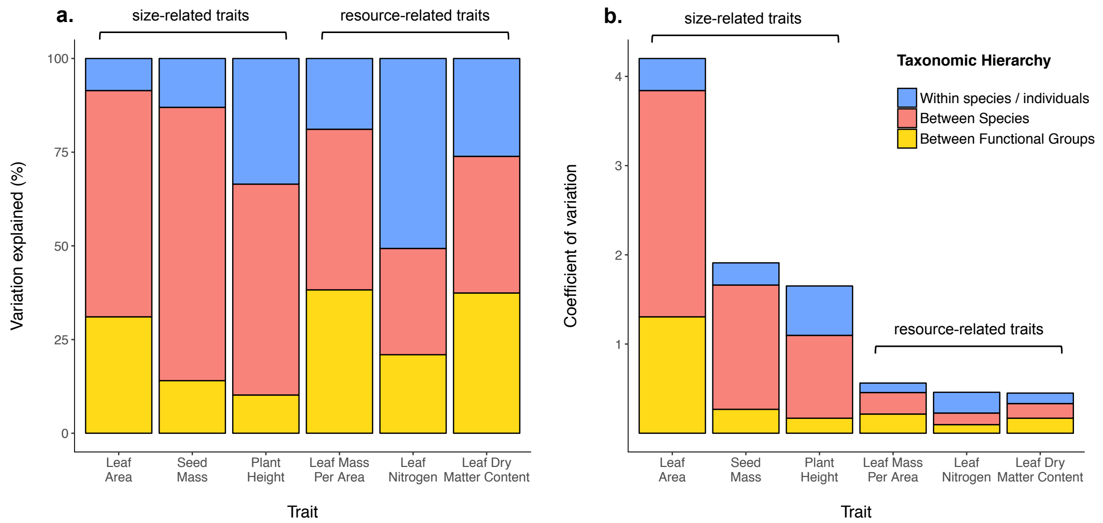

Supplementary Figure 18: Sources of trait variation for six plant traits in the tundra biome as in Fig. 3, , but only trait observations collected north of the Arctic Circle (66.5°N) or from collection locations with a mean annual temperature below 0°C. (a) Relative proportion of trait variation explained by functional group (deciduous shrubs, evergreen shrubs, graminoids, forbs; yellow), species (red) and within species (blue). (b) Total trait variation, represented by the coefficient of variation, and component sources of variation for each trait across the tundra biome.

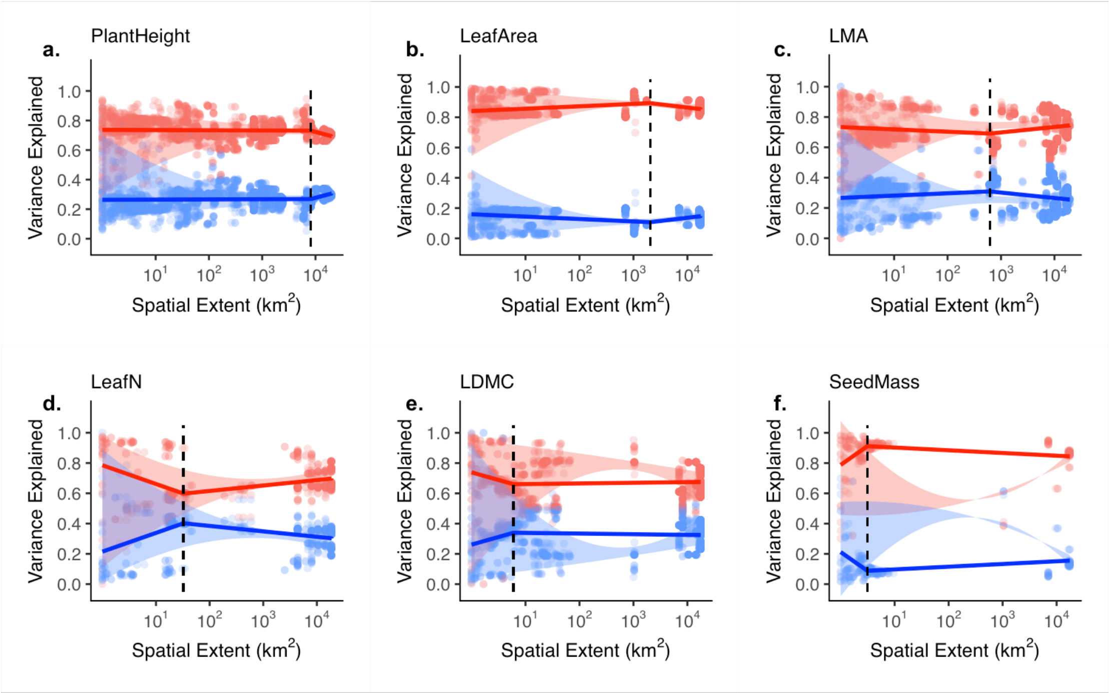

Supplementary Figure 19: Sources of trait variation across geographical scale and species richness as in Figure 4 and Supplementary Figure 6, but only trait observations collected north of the Arctic Circle (66.5°N) or from collection locations with a mean annual temperature below 0°C. Points indicate contribution of within-species (blue) and among-species (red) variation to total trait variation across trait sampling site combinations. Coloured lines indicate linear break point model fit with one break point. Dashed line indicates break point location.

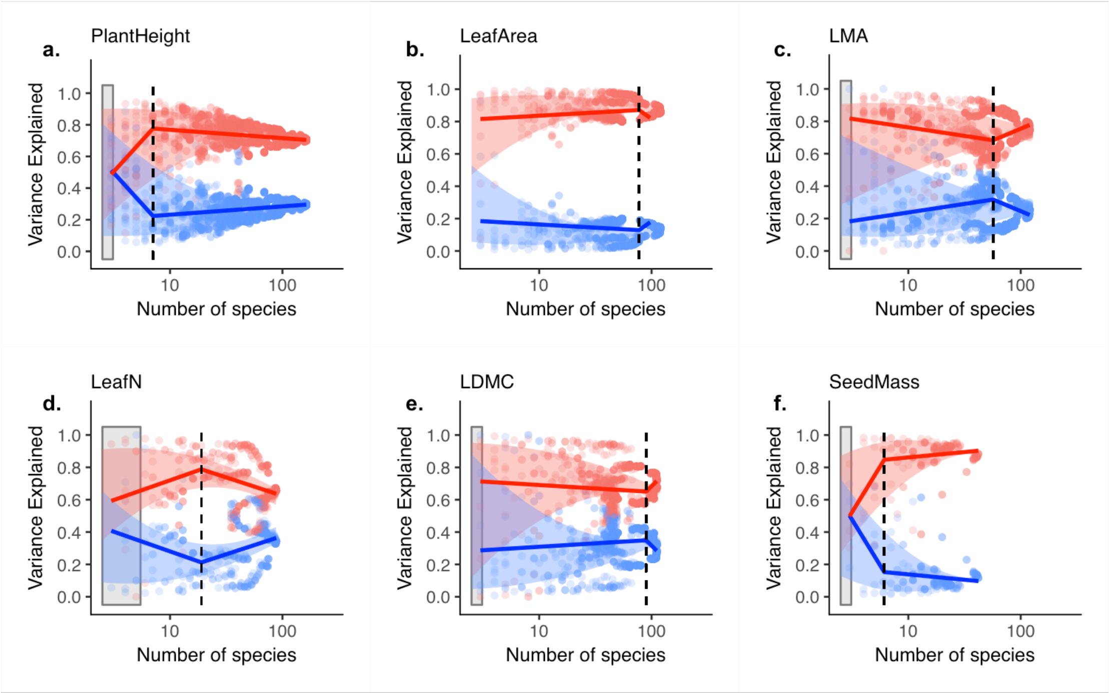

Supplementary Figure 20: Sources of trait variation across species richness as in Figure 4 and Supplementary Figure 6, but only trait observations collected north of the Arctic Circle (66.5°N) or from collection locations with a mean annual temperature below 0°C. Points indicate contribution of within-species (blue) and among-species (red) variation to total trait variation across trait sampling site combinations. Coloured lines indicate linear break point model fit with one break point. Dashed line indicates break point location. Grey box indicates where difference between among- and within-species variation is not significant (two-tailed t test, $P > 0.05$).

Supplementary Tables

Supplementary Table 1. Number of overall observations per trait, and those with latitude/longitude information for tundra species. The minimum and mean percent of species per site with at least one trait observation, and the minimum and mean percent cover (abundance) of species per site with at least one trait observation. The maximum % species and % cover is 100 for all traits.

Trait	Num. of observa-tions	Num. obs. w/ coordinates	% species (min)	% species (mean)	% cover (min)	% cover (mean)
Leaf Dry Matter Content	6552	6242	19	67.5	5	80.5
Leaf Area	9067	8820	20	82.1	10	90.0
Leaf N	4420	3860	29	72.1	30	86.8
Height	19396	18176	48	94.0	38	96.1
Seed Mass	2410	915	20	71.6	4	80.4
Specific Leaf Area	9817	8793	20	78.0	10	87.3

Supplementary References

1. Díaz, S. *et al.* The global spectrum of plant form and function. *Nature* **529**, 167–171 (2016).

2. Kattge, J. *et al.* TRY - a global database of plant traits. *Global Change Biology* **17**, 2905–2935 (2011).
