# Supplementary material for: Global plant trait relationships extend to the climatic extremes of the tundra biome
Source: Nat Commun. 2020 Mar 12;11:1351. doi: 10.1038/s41467-020-15014-4 (PMC7067758; doi:10.1038/s41467-020-15014-4)
Supplement: Supplementary file 3 — Reporting Summary [file 41467_2020_15014_MOESM3_ESM.pdf]

nature research

Corresponding author(s): Haydn Thomas

Last updated by author(s): Jan 27, 2020

# Reporting Summary

Nature Research wishes to improve the reproducibility of the work that we publish. This form provides structure for consistency and transparency in reporting. For further information on Nature Research policies, see Authors & Referees and the Editorial Policy Checklist .

## Statistics

For all statistical analyses, confirm that the following items are present in the figure legend, table legend, main text, or Methods section.

| n/a | Confirmed | |
|---|---|---|
| ☐ | ☒ | The exact sample size (*n*) for each experimental group/condition, given as a discrete number and unit of measurement |
| ☐ | ☒ | A statement on whether measurements were taken from distinct samples or whether the same sample was measured repeatedly |
| ☐ | ☒ | The statistical test(s) used AND whether they are one- or two-sided<br>*Only common tests should be described solely by name; describe more complex techniques in the Methods section.* |
| ☐ | ☒ | A description of all covariates tested |
| ☐ | ☒ | A description of any assumptions or corrections, such as tests of normality and adjustment for multiple comparisons |
| ☐ | ☒ | A full description of the statistical parameters including central tendency (e.g. means) or other basic estimates (e.g. regression coefficient) AND variation (e.g. standard deviation) or associated estimates of uncertainty (e.g. confidence intervals) |
| ☒ | ☐ | For null hypothesis testing, the test statistic (e.g. *F*, *t*, *r*) with confidence intervals, effect sizes, degrees of freedom and *P* value noted<br>*Give P values as exact values whenever suitable.* |
| ☒ | ☐ | For Bayesian analysis, information on the choice of priors and Markov chain Monte Carlo settings |
| ☐ | ☒ | For hierarchical and complex designs, identification of the appropriate level for tests and full reporting of outcomes |
| ☒ | ☐ | Estimates of effect sizes (e.g. Cohen's *d*, Pearson's *r*), indicating how they were calculated |

*Our web collection on statistics for biologists contains articles on many of the points above.*

## Software and code

Policy information about availability of computer code

| | |
|---|---|
| Data collection | No software was used for data collection |
| Data analysis | All analyses were conducted in R (v. 3.3.3). Code is available at github.com/hjdthomas/Tundra_trait_variation. |

For manuscripts utilizing custom algorithms or software that are central to the research but not yet described in published literature, software must be made available to editors/reviewers. We strongly encourage code deposition in a community repository (e.g. GitHub). See the Nature Research guidelines for submitting code & software for further information.

## Data

Policy information about availability of data

All manuscripts must include a data availability statement. This statement should provide the following information, where applicable:

- Accession codes, unique identifiers, or web links for publicly available datasets
- A list of figures that have associated raw data
- A description of any restrictions on data availability

All trait data compiled through the Tundra Trait Team are available at github.com/TundraTraitTeam/TraitHub, see Bjorkman et al. (2019b)30. Additional global trait data are available through the TRY database (try-db.org/TryWeb/dp.php)15.

# Field-specific reporting

Please select the one below that is the best fit for your research. If you are not sure, read the appropriate sections before making your selection.

☐ Life sciences ☐ Behavioural & social sciences ☒ Ecological, evolutionary & environmental sciences

# Ecological, evolutionary & environmental sciences study design

All studies must disclose on these points even when the disclosure is negative.

| | |
|---|---|
| Study description | We test whether 1) Trait expression among tundra species is constrained relative to global trait space, and whether trait relationships are maintained. 2) The contribution of within-species trait variation to total trait variation in the tundra is greater than the global average of 25%, and 3) The contribution of within-species trait variation to total trait variation is greater at local rather than at larger geographical scales.<br>We use the largest database of Arctic and alpine tundra plant traits ever compiled by combining 20,991 records from the TRY database with 30,616 records from the Tundra Trait Team (TTT), representing 89% of the tundra species pool. |
| Research sample | All tundra plant species with available trait data, and global plant species with available trait data for comparison. |
| Sampling strategy | In line with previous biome-scale assessments of tundra vegetation community change, we defined the tundra biome as the vegetated regions above treeline at high latitude and high altitude. Tundra species were identified as those present in sampling plots from two biome-scale experiments, the International Tundra Experiment (ITEX) and associated sites, and the sUMMITDiv network, or those present at trait collection sites with a mean annual temperature below 0oC.<br><br>We extracted trait data from the TRY9 3.0 database (available at www.try-db.org) for tundra species. We extracted traits of all tundra species from the TRY database regardless of location to maximize the capture of trait variation per species. We supplemented TRY data with additional trait data from the "Tundra Trait Team" (TTT) database. All species names from ITEX, TRY and TTT were matched to accepted names in The Plant List using the R package Taxonstand (v. 1.8) before merging the datasets.<br><br>We selected six plant traits: plant height (PH, maximum measured height), seed mass (SM, dry mass), leaf area per leaf (LA, fresh leaf area), leaf mass per area (LMA, ratio of leaf dry mass to fresh leaf area), leaf dry matter content (LDMC, ratio of leaf dry mass to fresh leaf mass), and leaf nitrogen (LN, nitrogen per unit leaf dry mass). These six traits are considered to represent fundamental dimensions of ecological strategy and are commonly measured in the tundra, thus maximizing trait coverage |
| Data collection | Trait data submitted to the TRY and TTT trait databases. |
| Timing and spatial scale | Trait data collected between 1980 - 2017.<br><br>Spatial scale is whole tundra biome, with global comparison |
| Data exclusions | TRY trait data were subjected to a multi-step cleaning process. Firstly, all values that did not represent individual measurements or species means were excluded. Secondly, we identified overlapping datasets within TRY and removed duplicate observations whenever possible. The following datasets were identified as having partially overlapping observations: GLOPNET – Global Plant Trait Network Database, The LEDA Traitbase, Abisko & Sheffield Database, Tundra Plant Traits Database, and KEW Seed Information Database (SID).<br><br>Thirdly, we removed duplicates within each TRY dataset (e.g., if a value is listed once as "mean" and once as "best estimate") by first calculating the ratio of duplicated values within each dataset, and then removing duplicates from datasets with more than 30% duplicated values. This cut-off was determined by manual evaluation of datasets at a range of thresholds. Datasets with fewer than 30% duplicated values were not cleaned in this way as any internally duplicate values were assumed to be true duplicates (i.e. two different individuals were measured and happened to have the same measurement value).<br><br>Finally, we removed all species mean observations from the "Niwot Alpine Plant Traits" database and replaced them with the original individual observations as provided by the trait collector (Marko J. Spasojevic) in order to ensure all trait measurements were for individuals.<br><br>Both datasets were checked for improbable values, with the goal of excluding likely errors or measurements with incorrect units but without excluding true extreme values. It was particularly important to avoid artificial reduction in the range of trait values in this study since we were explicitly interested in trait variation. We followed a series of data-cleaning steps, in each case estimating an error risk for a given observation (x) by calculating the difference between x and the mean (excluding x) of the group in question and then dividing by the standard deviation of the group.<br><br>We employed a hierarchical data cleaning method, because the standard deviation of a trait value is related to the mean and sample size. First, we checked individual records against the entire distribution of observations of that trait and removed any records with an error risk greater than eight (i.e., a value more than eight standard deviations away from the trait mean). For species that occurred in four or more unique datasets within TRY or TTT (i.e., different data contributors), we estimated a species mean per dataset and removed observations for which the species mean error risk was greater than three (i.e., the species mean of that dataset was more than three SD's away from the species mean across all datasets). For species that occurred in fewer than four unique datasets, we estimated a genus mean per dataset and removed observations in datasets for which the error risk based on the genus mean was greater than 3.5. Finally, we compared individual records directly to the distribution of values for that species. For species with fewer than four records we did not remove any values. For species with more than four records, we excluded values above an error risk Y, where Y was dependent on the number of records of that species and ranged from an error risk of 2.25 for species with fewer than 10 records to an error risk of four for species with more than 30 records. This procedure was performed on the complete tundra trait database – including species and traits not presented here. In total 3,515 observations (2.8%) were removed. In all cases, we visually checked the excluded values against the distribution of all observations for each species to ensure that our trait cleaning protocol |

| | |
|---|---|
| | was reasonable.<br><br>All trait observations with latitude/longitude information were mapped and checked for illogical values (e.g., falling in the ocean). These values were corrected from the original publications or by contacting the data contributor whenever possible. Where locations could not be verified, geo-referenced coordinates were removed and the trait data not included in scale-related analyses.<br><br>We excluded SSD from all analyses, except for comparisons with Diaz et al. (2016)6 (Supplementary Figures 9-11) since SSD had low collection coverage in the tundra and was available for too few species (n=52). |
| Reproducibility | All data and code made publicly available |
| Randomization | Species were allocated to four traditional functional groups - evergreen shrubs, deciduous shrubs, graminoids, and forbs - based on previous classification of ITEX species. |
| Blinding | Study is based on statistical analysis of existing dataset, so no blinding required. |

Did the study involve field work? ☐ Yes ☒ No

# Reporting for specific materials, systems and methods

We require information from authors about some types of materials, experimental systems and methods used in many studies. Here, indicate whether each material, system or method listed is relevant to your study. If you are not sure if a list item applies to your research, read the appropriate section before selecting a response.

## Materials & experimental systems

| n/a | Involved in the study | |
|---|---|---|
| ☒ | ☐ | Antibodies |
| ☒ | ☐ | Eukaryotic cell lines |
| ☒ | ☐ | Palaeontology |
| ☒ | ☐ | Animals and other organisms |
| ☒ | ☐ | Human research participants |
| ☒ | ☐ | Clinical data |

## Methods

| n/a | Involved in the study | |
|---|---|---|
| ☒ | ☐ | ChIP-seq |
| ☒ | ☐ | Flow cytometry |
| ☒ | ☐ | MRI-based neuroimaging |
